# Micronutrient status 2 years after bariatric surgery: a prospective nutritional assessment

**DOI:** 10.3389/fnut.2024.1385510

**Published:** 2024-05-14

**Authors:** Marianne Côté, Laurence Pelletier, Mélanie Nadeau, Léonie Bouvet-Bouchard, François Julien, Andréanne Michaud, Laurent Biertho, André Tchernof

**Affiliations:** ^1^Quebec Heart and Lung Institute – Laval University, Québec, QC, Canada; ^2^School of Nutrition, Faculty of Agricultural and Food Sciences, Laval University, Québec, QC, Canada; ^3^Department of Surgery, Faculty of Medicine, Laval University, Québec, QC, Canada

**Keywords:** sleeve gastrectomy, roux-en-Y gastric bypass, biliopancreatic diversion with duodenal switch, micronutrient deficiency, vitamin and mineral supplementation, severe obesity, bariatric surgery

## Abstract

**Background:**

Among commonly performed bariatric surgeries, biliopancreatic diversion with duodenal switch (BPD-DS) provides greater weight loss than Roux-en-Y gastric bypass (RYGB) or sleeve gastrectomy (SG), with sustained metabolic improvements. However, the risk of long-term nutritional deficiencies due to the hypoabsorptive component of BPD-DS hinders its widespread use.

**Objective:**

The aim of the study was to examine nutritional status over 2 years after BPD-DS, RYGB or SG.

**Methods:**

Patients were recruited in the REMISSION trial (NCT02390973), a single-center, prospective study. Out of 215 patients, 73, 48 and 94, respectively, underwent BPD-DS, RYGB or SG. Weight loss, micronutrient serum levels (including iron, calcium, parathormone, vitamins A, B12 and D), and nutritional supplementation were assessed over 2 years. Patients were supplemented according to the type of surgery and individual micronutrient level evolution.

**Results:**

At baseline, BPD-DS patients were younger than SG patients (*p* = 0.0051) and RYGB patients had lower body mass index (*p* < 0.001). Groups had similar micronutrient levels before surgery, with vitamin D insufficiency as the most prevalent nutritional problem (SG: 38.3%, RYGB: 39.9%, BPD-DS: 54.8%, *p* = 0.08). BPD-DS patients showed lower levels of iron, calcium and vitamin A than SG patients at 24 months. Groups had similar levels of vitamin D at 24 months. Prevalence of vitamin D, calcium, iron, vitamin A and vitamin B12 deficiency was similar among groups at 24 months. Rates of vitamin D insufficiency and iron deficiency were lower at 24 months than at baseline. Micronutrient intake was consistent with recommendations in groups post-surgery, but most BPD-DS patients took vitamin A and vitamin D supplement doses above initial recommendations.

**Conclusion:**

With appropriate medical and nutritional management, all surgeries led to similar rates of vitamin D, calcium, iron, vitamin A and vitamin B12 deficiencies at 24 months. However, initial vitamin A and vitamin D supplementation recommendations for BPD-DS patients should be revised upwards.

## 1 Introduction

Obesity is now recognized as a complex chronic disease characterized by an abnormal or excessive adiposity that impairs health (1). In severe obesity, lifestyle interventions are often ineffective to achieve sustainable weight loss and metabolic improvements that persist in the long term (2). Bariatric surgery is now recognized as one of the main pillars of obesity treatment (1). Indeed, it has been shown to be more effective than medication or nutritional counseling for glycemic control improvement of people living with severe obesity and type 2 diabetes (T2D) (3).

Among commonly performed surgical procedures to induce weight loss, sleeve gastrectomy (SG) is currently the most popular approach worldwide (4). It is a restrictive bariatric operation consisting of the resection of two-thirds of the greater curvature of the stomach while preserving the pylorus (5). SG leads to weight loss and comorbidities remission, but weight regain and T2D relapse is observed in some patients (6). Roux-en-Y gastric bypass (RYGB) is the second most frequently performed bariatric surgery worldwide (4). It is a mixed procedure, combining an important restrictive component and a small hypoabsorptive component with a 300 cm common limb (7). RYGB has been reported to have rates of T2D remission and complication similar to SG (8). Another type of mixed surgical approach offered to patients is the biliopancreatic diversion with duodenal switch (BPD-DS). This surgery includes a SG and a significant hypoabsorptive component, with a common limb of only 100 cm (5). BPD-DS leads to important and sustained weight loss with 80–90% T2D remission in the long term (6, 9). However, BPD-DS represented only 1.3% of the weight loss surgeries performed in 2021, mostly due to the perceived risk of long-term complications and technical complexity associated with the procedure (4, 10).

Many types of complications can occur after bariatric surgery, nutritional deficiency being one of them (11, 12). The nutritional risk associated with bariatric surgery differs according to the type of procedure and may influence the selection of a specific surgery for a given patient (10, 12). Restrictive procedures like SG reduce food intake and have a small impact on nutrient absorption resulting from gastric fundus resection. SG is associated with iron, folate, vitamin B12, vitamin D and calcium deficiency (12–15). Mixed procedures like RYGB and BPD-DS, in addition to reducing food intake, decrease the absorption of nutrients by bypassing their main absorption sites in the intestine. Therefore, high rates of liposoluble vitamins, minerals and trace element deficiencies have been observed after RYGB and BPD-DS (12–15). Because of its short common limb, BPD-DS is reported in the literature with the highest rates of micronutrient deficiencies when compared to SG or RYGB (14). Considering the nutritional risk associated with bariatric surgery, lifelong nutritional monitoring and supplementation is recommended (12, 14, 15), and low adherence to supplementation recommendations represents an additional risk component after surgery (12).

Nutritional management of bariatric patients is complex because nutritional deficiencies have been reported even before surgery (13). The presence of nutritional problems in obesity seems paradoxical in a context of high caloric intake but can be explained by multifactorial causes. Consumption of energy-dense food with a low-nutrient density may contribute to micronutrient deficiencies (13, 16). Also, low-grade chronic inflammation, present in the obesity state, may affect micronutrient absorption such as iron (13, 16). Furthermore, increased adiposity may impact micronutrient status, especially for nutrients that are soluble in adipose tissue like vitamin D (17). Vitamin D appears to be the most frequent micronutrient deficiency observed in patients prior to bariatric surgery, but iron and vitamin A deficiencies are also frequent in that population (16, 18, 19). Addressing these deficiencies before weight loss surgery is essential to avoid adverse nutritional outcomes during follow-up (18, 19).

Although the nutritional risk of SG and RYGB is well described in the literature, most of the available data are from retrospective studies. Only a few studies have examined the risk associated with BPD-DS on micronutrient status. Furthermore, the literature on micronutrient status after bariatric surgery is difficult to assess because reference values used to determine deficiency differ considerably among studies and information on vitamin and mineral intake is often variable or unreported. Finally, prospective studies comparing micronutrient status and deficiencies following SG, RYGB and BPD-DS are scarce. To gain a better understanding of the nutritional risk associated with commonly performed surgeries, the objective of the study was to examine micronutrient levels, micronutrient deficiencies and adherence to initial vitamin and mineral supplementation recommendations in a prospective design over 2 years after SG, RYGB or BPD-DS.

## 2 Methods

### 2.1 Study participants

To compare the effect of SG, RYGB and BPD-DS on nutritional status, 215 participants were examined in a 5-year, single-center, prospective design. This study is part of the REMISSION trial (NCT02390973) evaluating T2D remission and metabolic recovery following SG, RYGB or BPD-DS. Although BPD-DS is less frequently performed compared to RYGB or SG, it was included because it has been shown as the most effective procedure for weight loss and T2D resolution. Of the 215 participants, 193 have completed the 2-year follow-up while 7 participants dropped out and 15 had not yet completed their 24 months follow-up at the time this study was conducted. The follow-up of the cohort is still ongoing and continues up to 5 years. Inclusion criteria were the following: patients with a body mass index (BMI) ≥35 kg/m^2^ living with T2D who required surgery and met the NIH Guidelines for bariatric surgery (20); patients who had 1 year of follow-up completed in January 2022. Exclusion criteria were general contra-indications for bariatric surgery, a BMI <35 kg/m^2^, age under 18 or over 60 years, abnormal bowel habits including irritable bowel syndrome, pregnancy, cirrhosis or albumin deficiency and previous bariatric surgery. The Research Ethics Committee of the *Institut universitaire de cardiologie et de pneumologie de Québec – Université Laval* (IUCPQ-ULaval) approved this study (#2015–2,466, 21,160). All participants provided informed consent to participate in the study.

### 2.2 Surgical procedures

All surgeries were performed laparoscopically. A 250 cm^3^ vertical SG was created with a 34–44 French Bougie starting 7–8 cm from the pylorus (21). The RYGB was performed by creating a 30–50 cm^3^ proximal gastric pouch connected to the proximal small intestine by bypassing the first 100 cm. A 100-cm alimentary limb was then anastomosed to the gastric pouch, with a 300-cm common channel (22). For the BPD-DS, a 250 cm^3^ SG was created and the duodenum was transected about 4 cm distal from the pylorus and anastomosed to a 250-cm alimentary limb, with a 100-cm common channel (23).

### 2.3 Study design

Participants received preoperative and postoperative care by a multidisciplinary team composed of bariatric surgeons, bariatric nurses, dieticians, social workers and other health professionals if needed. They were followed longitudinally at 4, 8, 12 and 24 months after surgery to assess anthropometric measurements, medical evaluation and evolution of comorbidities. Nutritional status was evaluated according to micronutrient serum levels including vitamin A, vitamin B12 and vitamin D (25-OH-D), folate, calcium, parathormone (PTH), sodium, potassium, chloride, magnesium, phosphorus, iron, ferritin, transferrin and hemoglobin. Albumin and prealbumin serum levels were also assessed. Assays were performed at the laboratory of the IUCPQ-ULaval. Nutritional deficiencies were determined according to blood level reference values used for clinical practice at the IUCPQ-ULaval (Supplementary Table S1). Suboptimal vitamin D status was separated in two categories: insufficiency (levels of 25-OH-D between 30–49 nmol/L) and deficiency (levels of 25-OH-D below 30 nmol/L).

Participants received vitamin D and multivitamin supplementation 3 to 6 months before surgery. Other micronutrients were supplemented if deficiencies were present at baseline to address them before surgery. Daily supplementation was then started 1 month after surgery according to the type of procedure received. The daily recommendations for SG patients were vitamin D 1000 IU and 1 multivitamin tablet (Pfizer Centrum Forte; contains, among other nutrients, vitamin D 600 IU, vitamin A 1000 IU, vitamin B12 20 μg, folic acid 400 μg, calcium 200 mg, iron 10 mg). The daily recommendations for RYGB patients were 2 multivitamin tablets, vitamin D 2000 IU, calcium carbonate 1,000 mg, ferrous sulfate 300 mg and vitamin B12 1,200 μg. The daily recommendations for BPD-DS patients were 2 multivitamin tablets, vitamin D 30000 IU, calcium carbonate 1,000 mg, ferrous sulfate 300 mg and vitamin A 30,000 IU. Supplements were adjusted during follow-up according to individual blood levels. Adherence to vitamin and mineral initial supplementation recommendations was evaluated for all patients according to supplement intake assessed in the pharmacy prescription and double-checked with patient self-reported intake at each follow-up visit. Vitamin and mineral intakes were characterized as being below, on or above target when supplement intake was inferior, equal or greater than surgery-specific initial recommendations, respectively.

### 2.4 Statistical analyses

Numerical data are presented as mean ± standard deviation when normally distributed and median ± interquartile range otherwise. Results of categorical variables are presented as percentages. Repeated-measures ANOVA, adjusted for age and baseline BMI, was performed to examine changes in nutrient levels 0 to 24 months after SG, RYGB or BPD-DS. Differences among surgical procedures were assessed with Tukey-HSD multiple comparisons test at each time point. To compare surgical groups on baseline categorical variables, prevalence of nutritional deficiencies and adherence to vitamin and mineral initial supplementation recommendations, Chi-squared test or Fisher’s exact test were performed according to the number of observations. Changes in adherence to initial supplementation recommendations between 12 and 24 months were assessed with generalized linear mixed-effects models for binomial variables and with generalized estimating equations for multinomial variables. The study was sufficiently powered to detect a difference in the main nutritional variables or at least 25% between surgical groups, with 80% power and α <0.05. A *p*-value below 0.05 was considered statistically significant. Statistical analyses were performed with RStudio version 2022.12.0.353 (Posit Software, PBC, Boston, MA).

## 3 Results

**Figure 1:**
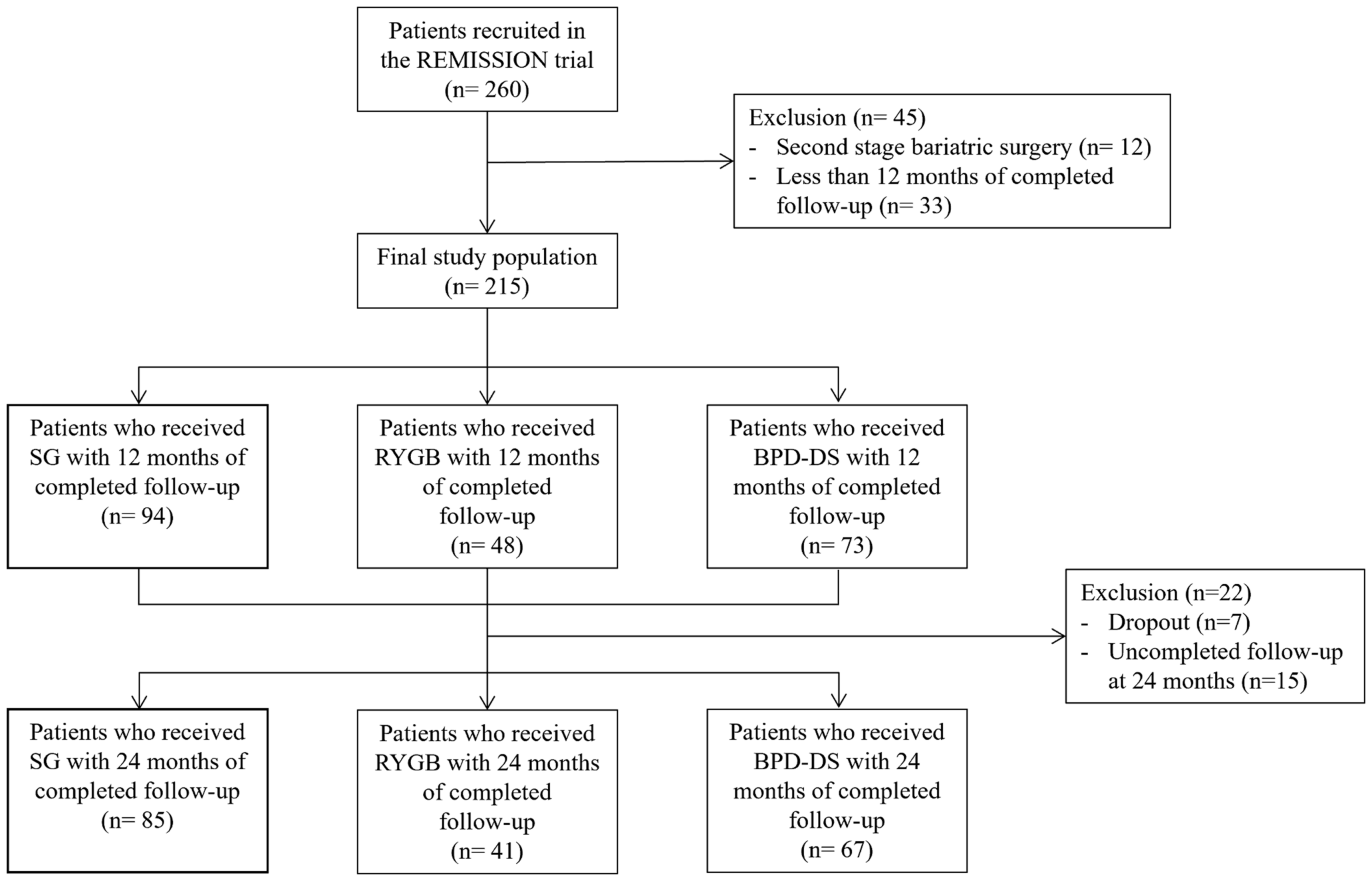
Flowchart of the study participants. Sleeve Gastrectomy (SG), Roux-en-Y Gastric Bypass (RYGB), Biliopancreatic Diversion with Duodenal Switch (BPD-DS).

Of the 215 participants recruited, 94 patients underwent SG, 48 underwent RYGB and 73 underwent BPD-DS (Figure 1). BPD-DS patients were younger than SG patients (*p* < 0.05) (Table 1). The BPD-DS group had the highest preoperative weight and BMI compared to the SG and RYGB groups (*p* < 0.05). Female and male ratios were comparable in each group. All participants had T2D and the three groups had high rates of hypertension and dyslipidemia prior to surgery.

**Table 1:** Preoperative characteristics of the study participants.

Variables	SG			RYGB			BPD-DS			*p*-value
*n*	94			48			73			
Sex (F:M)	50:44			29:19			37:36			0.5647
Age (years)	52.3 ± 10.9			51.7 ± 12.3			46.8 ± 12.0^a^			0.005625
Weight (kg)	123.1 ± 21.6			111.0±14.8^a^			135.6 ± 18.2^a,b^			< 0.001
BMI (kg/m^2^)	44.3 ± 5.4			40.1±3.1^a^			47,0 ± 4.9^a,b^			< 0.001
Diabetes (%)	94 (100)			48 (100)			73 (100)			1
Hypertension (%)	74 (78.7)			34 (70.8)			59 (81.9)			0.3464
Dyslipidemia (%)	81 (86.2)			40 (83.3)			59 (80.8)			0.6399

Data analyzed with ANOVA or chi-square tests as appropriate. Results are presented as mean ± standard deviation when normally distributed and median ± interquartile range otherwise. a: different from SG (*p* < 0.05), b: different from RYGB (*p* < 0.05) after Tukey-HSD multiple comparisons. BMI, Body Mass Index.

**Figure 2:**
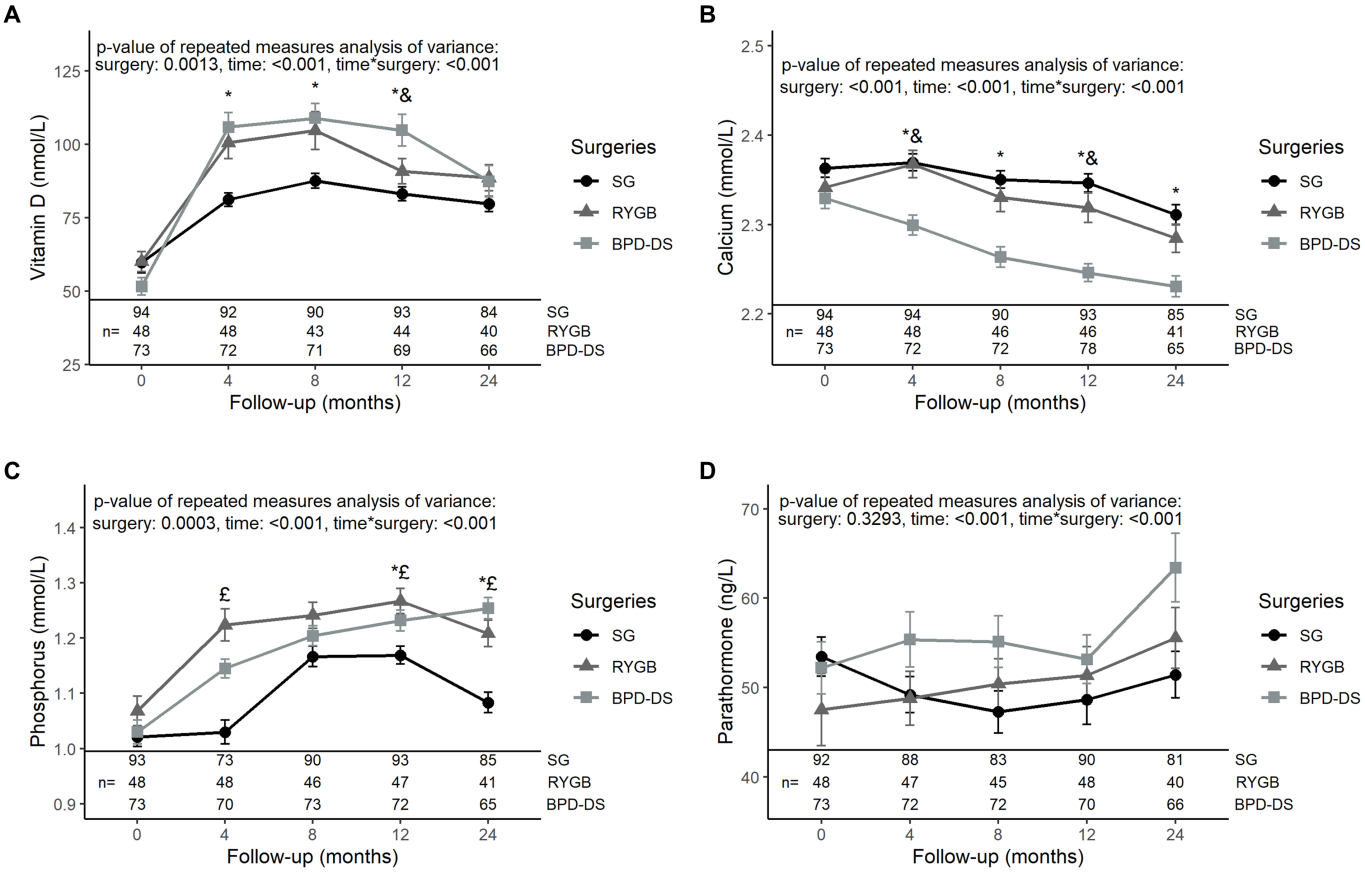
Serum levels of phosphocalcium metabolism markers in patients receiving SG, RYGB or BPD-DS from 0 to 24 months. **(A)** Vitamin D, **(B)** calcium, **(C)** phosphorus and **(D)** parathormone 0 to 24 months post-operation for SG, RYGB and BPD-DS groups. Repeated measures analysis of variance are adjusted for age and baseline BMI. Statistical tests were performed on log-transformed values for **(A,B,D)**. *: difference between SG and BPD-DS (*p* < 0.05), &: difference between RYGB and BPD-DS (*p* < 0.05), £: difference between SG and RYGB (*p* < 0.05) after Tukey-HSD multiple comparisons. Data are presented as means and standard error of the mean (SEM). SG, sleeve gastrectomy, RYGB, Roux-en-Y gastric bypass, BPD-DS, biliopancreatic diversion with duodenal switch.

All three groups had similar levels of vitamin D, calcium, phosphorus and PTH at baseline (Figure 2). Vitamin D levels of the BPD-DS group were higher than the SG group at 4, 8 and 12 months (*p* < 0.01) and were higher than the RYGB group only at 12 months (*p* < 0.01) (Figure 2A). However, at 24 months, vitamin D levels were similar among groups and were higher compared to baseline (*p* < 0.05). For calcium levels, BPD-DS patients had lower serum levels than the SG group from 4 to 24 months after surgery (*p* < 0.001 for all time points) (Figure 2B). BPD-DS and RYGB groups were only different at 4 and 12 months (*p* < 0.001 and *p* < 0.05 respectively), with the BPD-DS group having the lowest calcium levels. Globally, calcium levels decreased significantly during the entire follow-up for all groups. Phosphorus levels increased significantly during follow-up for all groups but started to decrease between 12 and 24 months for SG and RYGB (*p* < 0.001 and *p* < 0.05 respectively) (Figure 2C). At 12 and 24 months, the SG group had the lowest phosphorus levels of all surgical groups (*p* < 0.05). PTH levels of the RYGB and BPD-DS groups significantly increased in the postoperative period (*p* < 0.01) and there was no difference among groups for PTH levels during follow-up (Figure 2D).

**Figure 3:**
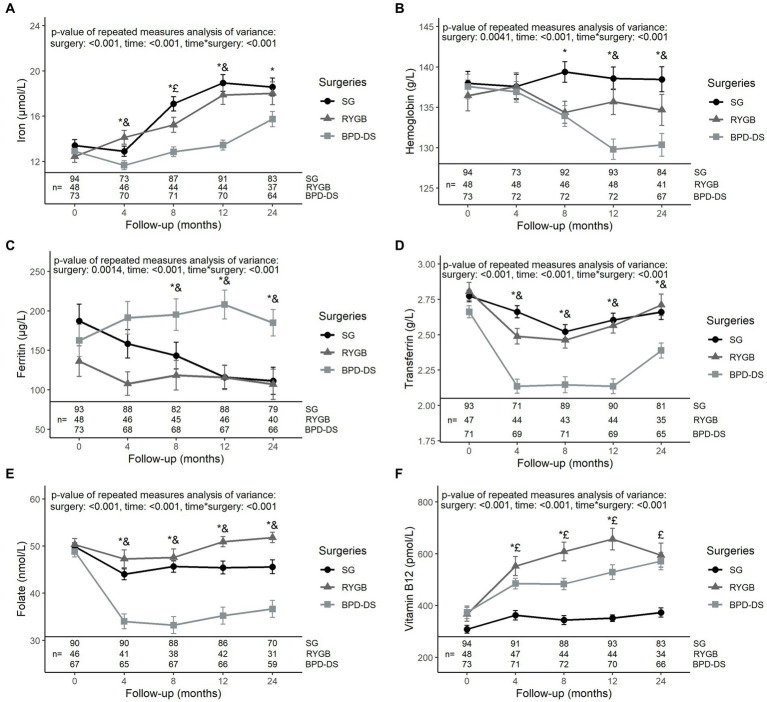
Serum levels of iron status markers in patients receiving SG, RYGB or BPD-DS from 0 to 24 months. **(A)** Iron, **(B)** hemoglobin, **(C)** ferritin, **(D)** transferrin, **(E)** folate and **(F)** vitamin B12 0 to 24 months post-operation for SG, RYGB and BPD-DS groups. Repeated measures analysis of variance are adjusted for age and baseline BMI. Statistical tests were performed on log-transformed values for **(F)**. ^*^: difference between SG and BPD-DS (*p* < 0.05), &: difference between RYGB and BPD-DS (*p* < 0.05), £: difference between SG and RYGB (*p* < 0.05) after Tukey-HSD multiple comparisons. Data are presented as means and standard error of the mean (SEM). SG, sleeve gastrectomy, RYGB, Roux-en-Y gastric bypass, BPD-DS, biliopancreatic diversion with duodenal switch.

The three surgical groups had similar levels of iron, hemoglobin, ferritin, transferrin, folate and vitamin B12 at baseline (Figure 3). Iron levels increased significantly after surgery in all groups, with BPD-DS having lower levels compared to SG from 4 to 24 months (*p* < 0.05 at all time points) (Figure 3A). Hemoglobin levels remained stable for the SG and RYGB groups during follow-up (Figure 3B). For BPD-DS, hemoglobin levels decreased in post-op so that, at 12 and 24 months, levels were significantly lower than the SG and RYGB groups. Ferritin levels of the SG group decreased during the postoperative period (*p* < 0.001) (Figure 3C). For BPD-DS participants, ferritin levels remained stable during follow-up and were significantly higher than SG and RYGB from 8 to 24 months. Transferrin levels of BPD-DS participants decreased over time to significantly lower values than the two other surgical groups from 4 to 24 months (Figure 3D). The same pattern was observed for folate levels (Figure 3E). For all groups, vitamin B12 levels increased over time (Figure 3F). However, vitamin B12 levels of the SG group remained significantly lower compared to RYGB or BPD-DS groups throughout the postoperative period.

Electrolytes, magnesium, vitamin A, albumin and prealbumin levels were also evaluated and are presented in Table 2. Sodium levels of the SG group were the highest at baseline (*p* < 0.03), though differences were minor. Sodium evolution of the three groups remained similar after surgery. For potassium, BPD-DS had higher levels compared to RYGB at baseline (*p* = 0.04). Then, at 4 and 8 months, the BPD-DS group had significantly lower levels compared to the other surgical groups but at 12 months the BPD-DS group was only different from the SG group (*p* < 0.01). Potassium levels became similar for all groups at 24 months. The BPD-DS group had higher chloride levels than the SG group from 4 to 12 months and higher levels than the RYGB group only at 12 months. There was no difference in magnesium levels among surgical groups during the entire follow-up. Vitamin A levels were similar for all groups at baseline. After surgery, vitamin A levels of the BPD-DS group decreased to become lower than the other surgical groups from 4 to 12 months and lower than RYGB only at 24 months. Albumin levels were similar among groups at baseline, but BPD-DS participants reached lower levels than SG participants 4 months after surgery. At 8 and 12 months, BPD-DS participants had the lowest levels of all three groups, but no difference was observed among groups at 24 months. Prealbumin levels of BPD-DS participants were the lowest of all three groups from baseline to 8 months after surgery. Then, from 12 months, levels of prealbumin in the BPD-DS group increased so that they were only different from SG at 12 months and similar to all groups at 24 months.

**Table 2:** Evolution of electrolytes, magnesium, vitamin A, albumin and prealbumin serum levels from 0 to 24 months.

Nutrient	Group	Baseline	4 months	8 months	12 months	24 months	*p*-value of repeated measures analysis of variance		
							Surgery	Time	Time*surgery
Sodium (mmol/L)	SG	140 ± 2	141 ± 2	141 ± 1	141 ± 2	141 ± 2			
	RYGB	139 ± 2^a^	141 ± 2	141 ± 2	141 ± 2	140 ± 2	<0.001	0.1689	<0.001
	BPD-DS	139 ± 2^a^	141 ± 2	141 ± 2	141 ± 1	141 ± 2			
Potassium (mmol/L)	SG	3.9 ± 0.3	4.0± 0.3	4.1± 0.3	4.1± 0.3	4.0± 0.3			
	RYGB	3.9 ± 0.3	4.0± 0.3	4.0± 0.3	4.0± 0.3	4.0± 0.4	<0.001	0.004	<0.001
	BPD-DS	4.0 ± 0.3^b^	3.8±0.4^a,b^	3.8±0.5^a,b^	3.9±0.3^a^	4.0± 0.3			
Chloride (mmol/L)	SG	102 ± 3	104±3	104±3	105±3	105±3			
	RYGB	103 ± 3	105±3	105±3	105±3	105±3	<0.001	0.0014	<0.001
	BPD-DS	102 ± 3	106 ± 3^a^	106±3^a^	106±3^a,b^	106±3			
Magnesium (mmol/L)	SG	0.78 ± 0.09	0.80 ± 0.07	0.81±0.08	0.82±0.09	0.83±0.07			
	RYGB	0.79 ± 0.09	0.81 ± 0.06	0.82±0.07	0.83±0.06	0.83±0.07	<0.001	0.2739	<0.001
	BPD-DS	0.78 ± 0.09	0.79 ± 0.09	0.80±0.08	0.80±0.08	0.81±0.08			
Vitamin A (μmol/L)	SG	2.0±0.5	1.7±0.5	1.9±0.4	1.9±0.5	2.1±0.5			
	RYGB	2.0±0.5	1.6±0.5	1.7±0.4^a^	1.8±0.4	1.9±0.5	<0.001	<0.001	<0.001
	BPD-DS	1.9±0.5	1.3±0.4^a,b^	1.4±0.4^a,b^	1.4 ± 0.4^a,b^	1.7±0.5^a^			
Albumin (g/L)	SG	42±5	42±5	41±3	41±4	41±4			
	RYGB	41±4	42±5	40±4	41±4	40±4	<0.001	<0.001	<0.001
	BPD-DS	41±4	40±6^a^	38±4^a,b^	38±4^a,b^	40±4			
Prealbumin (mg/L)	SG	279±66	249±48	267±57	267±90	301±72			
	RYGB	270 ± 61	240±61	250±51	243±76	276±51	<0.001	<0.001	<0.001
	BPD-DS	261 ± 50^a,b^	198±41^a,b^	197±41^a,b^	215±65^a^	263±50			

Repeated measures analysis of variance are adjusted for age and baseline BMI. a: different from SG (*p* < 0.05), b: different from RYGB (*p* < 0.05) after Tukey-HSD multiple comparisons. SG: sleeve gastrectomy, RYGB: Roux-en-Y gastric bypass, BPD-DS: biliopancreatic diversion with duodenal switch. Number of participants were as follows for SG, RYGB and BPD-DS respectively: baseline, 94,48 and 73; 4 months, 94, 48 and 72; 8 months, 94,47 and 73; 12 months, 94, 48 and 73; 24 months, 85, 41 and 67.

To evaluate the nutritional risk of SG, RYGB and BPD-DS, nutritional deficiency rates of iron, calcium, PTH, vitamin D, vitamin A and vitamin B12 were assessed. Prevalence of nutritional deficiencies and high PTH are presented in Table 3. Before surgery, vitamin D insufficiency was the most prevalent nutritional problem in all three groups. Iron and vitamin D deficiency were also prevalent in all groups at baseline. Iron deficiency rates remained high 4 months after surgery, with a tendency for more deficiencies in the BPD-DS group (*p* = 0.0537). Then, they decreased during the rest of the follow-up to a rate of 5–6% for all groups at 24 months. The prevalence of calcium deficiency was low at baseline and during the entire follow-up. More BPD-DS patients had calcium deficiency at 8 months, but at 24 months only a tendency for more deficiency in the BPD-DS group was observed (*p* = 0.0615). Rates of high PTH were low in all groups at baseline and during the entire follow-up. No patients had high PTH at 24 months. The prevalence of vitamin D insufficiency and deficiency decreased between baseline and 24 months without statistical differences among groups. Rates of vitamin D insufficiency remained low at 12 and 24 months and vitamin D deficiency was nearly absent at the end of the follow-up in all groups. Vitamin A and vitamin B12 deficiency was almost absent in all groups during the entire follow-up.

**Table 3:** Prevalence of nutritional deficiencies and high parathormone from 0 to 24 months after surgery.

Nutritional indicator	Group	Baseline	4 months	8 months	12 months	24 months
		*n* (%)	*n* (%)	*n* (%)	*n* (%)	*n* (%)
Iron deficiency	SG	19 (20.9)	12 (13.5)	8 (9.2)	4 (4.4)	2 (5.4)
	RYGB	10 (20.8)	8 (17.4)	2 (4.6)	3 (6.8)	2 (5.4)
	BPD-DS	12 (16.4)	20 (28.6)	11 (15.5)	5 (7.1)	4 (6.3)
Calcium deficiency	SG	1 (1.1)	0 (0)	0 (0)	1 (1.2)	1 (1.2)
	RYGB	1 (2.1)	0 (0)	0 (0)	0 (0)	0 (0)
	BPD-DS	1 (1.4)	0 (0)	5 (6.9)^a^	4 (5.6)	5 (7.7)
High parathormone	SG	2 (2.2)	1 (1.1)	2 (2.4)	3 (3.3)	0 (0)
	RYGB	2 (4.2)	0 (0)	0 (0)	0 (0)	0 (0)
	BPD-DS	4 (5.5)	3 (4.2)	3 (4.2)	4 (5.7)	0 (0)
Vitamin D insufficiency	SG	35 (38.8)	4 (4.3)	1 (1.1)	5 (5.4)	7 (8.2)
	RYGB	19 (39.6)	1 (2.1)	2 (4.7)	5 (11.1)	3 (7.5)
	BPD-DS	40 (54.8)	2 (2.8)	2 (2.8)	1 (1.4)	9 (13.6)
Vitamin D deficiency	SG	12 (12.8)	1 (1.1)	1 (1.1)	0 (0)	0 (0)
	RYGB	3 (6.3)	0 (0)	2 (4.7)	0 (0)	0 (0)
	BPD-DS	14 (19.2)	1 (1.4)	2 (2.8)	0 (0)	1 (1.4)
Vitamin A deficiency	SG	0 (0)	0 (0)	0 (0)	0 (0)	0 (0)
	RYGB	0 (0)	0 (0)	0 (0)	0 (0)	0 (0)
	BPD-DS	1 (1.4)	3 (4.3)	0 (0)	1 (1.4)	1 (1.4)
Vitamin B12 deficiency	SG	4 (4.3)	1 (1.1)	0 (0)	0 (0)	0 (0)
	RYGB	0 (0)	0 (0)	0 (0)	0 (0)	1 (2.3)
	BPD-DS	1 (1.4)	0 (0)	0 (0)	0 (0)	0 (0)

Data analyzed with Fisher’s exact ou chi-square tests as appropriate. a: different from SG and RYGB (*p* < 0.05) after Fisher’s exact test. Deficiencies are determined according to references values presented in Supplementary Table S1.

Adherence to vitamin and mineral initial supplementation recommendations at baseline, 12 and 24 months after surgery are presented for all three groups in Table 4. At baseline, supplementation was not yet initiated for most patients. Overall, results show high adherence to initial supplementation recommendations during the first 24 months after surgery, with most patients taking adequate supplementation to prevent deficiencies. Most patients in all groups were mostly on target for vitamin B12 (>80%), iron (>50%), calcium (>60%), and vitamin A only for SG and RYGB (>80%). However, at 24 months, more than 50% of BPD-DS patients took higher doses of vitamin A and vitamin D compared to recommendations, while most SG and RYGB patients took less vitamin D than targeted doses. As for multivitamin, SG participants were mostly on target (>70%), and the RYGB and BPD-DS groups were mostly below target (>60%). BPD-DS patients were statistically given more vitamin D than RYGB and SG patients at 12 and 24 months. More RYGB patients were below the target dose for vitamin D compared to the two other surgeries at 24 months. At 12 and 24 months, the SG group had statistically more patients on target for multivitamin supplementation compared to BPD-DS and RYGB, which were mostly in the below-target category for this supplement. At 24 months, more RYGB patients were in the below-target category for iron and calcium compared to BPD-DS patients who were in higher proportions in the on-target category for these two supplements. More BPD-DS patients took calcium doses above target compared to RYGB at 24 months. Rates of adherence to initial recommendations remained stable during follow-up for all supplements except for calcium. BPD-DS and RYGB patients were mostly on target for calcium at 12 months, with a significant proportion who went above target at 24 months for BPD-DS, while RYGB patients went mostly below target.

**Table 4:** Adherence to vitamin and mineral initial supplementation recommendations of SG, RYGB and BPD-DS patients 12 and 24 months after surgery.

Supplement	Group	Baseline			12 months			24 months		
		Below target	On target	Above target	Below target	On target	Above target	Below target	On target	Above target
		*n* (%)	*n* (%)	*n* (%)	*n* (%)	*n* (%)	*n* (%)	*n* (%)	*n* (%)	*n* (%)
Vitamin D	SG	18 (19.6)	62 (67.4)	12 (13.0)	33 (35.1)	54 (57.4)	7 (7.4)^c^	39 (44.8)	39 (44.8)	9 (10.3)^c^
	RYGB	46 (95.8)	0 (0)	2 (4.2)	23 (47.0)	22 (45.8)	3 (6.3)^c^	26 (60.5)	10 (23.3)^a,b^	7 (16.3)^c^
	BPD-DS	73 (100.0)	0 (0)	0 (0)	15 (20.5)	20 (27.4)	38 (52.1)	14 (20.3)	16 (23.2)	39 (56.5)
Vitamin A	SG	0 (0)	87 (92.6)	7 (7.4)	0 (0)	85 (90.4)	9 (9.6)	0 (0)	81 (93.1)	6 (6.9)
	RYGB	0 (0)	48 (100.0)	0 (0)	0 (0)	44 (91.7)	4 (8.3)	0 (0)	38 (88.4)	5 (11.6)
	BPD-DS	73 (100.0)	0 (0)	0 (0)	5 (6.8)	33 (45.2)	35 (47.9)	8 (11.6)	24 (34.8)	37 (53.6)
Vitamin B12	SG	0 (0)	88 (93.6)	5 (5.3)	0 (0)	89 (94.7)	5 (5.3)	0 (0)	80 (92.0)	7 (8.0)
	RYGB	45 (93.8)	3 (6.3)	0 (0)	7 (14.6)	41 (85.4)	0 (0)	8 (18.6)	35 (81.4)	0 (0)
	BPD-DS	0 (0)	69 (94.5)	4 (5.5)	0 (0)	70 (95.9)	3 (4.1)	0 (0.0)	66 (95.7)	3 (4.3)
Iron	SG	0 (0)	84 (89.4)	10 (10.6)	0 (0)	85 (90.4)	10 (10.6)	0 (0)	81 (93.1)	6 (6.9)
	RYGB	36 (75.0)	9 (18.8)	3 (6.3)	6 (12.5)	40 (83.3)	2 (4.2)	12 (27.9)	24 (55.8)^a^	7 (16.3)
	BPD-DS	62 (84.9)	9 (12.3)	2 (2.7)	7 (9.6)	53 (72.6)	13 (17.8)	8 (11.6)	47 (68.1)	14 (20.3)
Calcium	SG	0 (0)	91 (96.8)	3 (3.2)	0 (0)	91 (96.8)	3 (3.2)	0 (0)	78 (89.7)	9 (10.3)
	RYGB*	46 (95.8)	2 (4.2)	0 (0)	9 (18.8)	37 (77.1)	2 (4.2)	15 (34.9)	26 (60.5)^a^	2 (4.7)^c^
	BPD-DS*	73 (100.0)	0 (0)	0 (0)	7 (9.6)	59 (80.8)	7 (9.6)	8 (11.6)	43 (62.3)	18 (26.1)
Multivitamin	SG	15 (16.0)	79 (84.0)	0 (0)	15 (16.0)	79 (84.0)^a^	0 (0)	22 (25.3)	63 (72.4)^a^	2 (2.3)
	RYGB	47 (97.9)	1 (2.1)	0 (0)	29 (60.4)	19 (39.6)^b^	0 (0)	27 (62.8)	16 (37.2)^b^	0 (0)
	BPD-DS	72 (98.6)	1 (1.4)	0 (0)	46 (63.0)	27 (37.0)	0 (0)	44 (63.8)	24 (34.8)	1 (1.4)

Below target: patients taking supplement doses below initial daily recommendations. On target: patients taking supplement doses according to the initial daily recommendations. Above target: patients taking supplement doses above initial daily recommendations. Daily recommendations for vitamin and mineral supplementation are presented in the Methods. a: different from BPD-DS (*p* < 0.05). b: different from SG (*p* < 0.05). c: different from BPD-DS between above target and cumulative proportions of on target and below target (*p* < 0.05). *: adherence significantly different between 12 and 24 months (*p* < 0.05). SG: sleeve gastrectomy, RYGB: Roux-en-Y gastric bypass, BPD-DS: biliopancreatic diversion with duodenal switch. Number of participants were as follows for SG, RYGB and BPD-DS respectively: baseline, 94,48 and 73; 4 months, 94, 48 and 72; 8 months, 94,47 and 73; 12 months, 94, 48 and 73; 24 months, 85, 41 and 67.

## 4 Discussion

The aim of the study was to examine micronutrient levels, micronutrient deficiencies and adherence to vitamin and mineral initial supplementation recommendations to characterise nutritional status over 2 years after SG, RYGB or BPD-DS. Current literature on bariatric procedures and micronutrient deficiencies mainly examined SG and RYGB in retrospective studies. To our knowledge, this is the first study to compare micronutrient status and deficiencies of patients undergoing SG, RYGB or BPD-DS in a prospective design.

### 4.1 Nutritional deficiencies

For all nutrients, rates of deficiency were low and similar among groups. These results differ from literature where BPD-DS is reported to cause higher deficiency rates compared to other procedures, mostly for liposoluble vitamins (24, 25). Yet, some studies reported similar or lower rates of vitamin D, vitamin B12 or iron deficiency for SG compared to RYGB (8, 26, 27). Vitamin D insufficiency was the most prevalent nutritional problem after surgery and only one patient in the sample had vitamin D deficiency at 24 months. Previous studies reported vitamin D insufficiency or deficiency as the most prevalent nutritional problem after bariatric surgery, with prevalence rates higher than our results (25, 28, 29). Less than 10% of patients from all groups suffered from iron or calcium deficiency and 2% or less of the patients presented a vitamin A and vitamin B12 deficiency or high PTH after 2 years. Prospective studies on SG, RYGB or BPD-DS showed higher rates of micronutrient deficiencies after bariatric surgery (25, 30, 31). Other retrospective studies from our group presented comparably low rates after BPD-DS (9, 32). Because of the restrictive component reducing food intake and the hypoabsorptive component bypassing major absorption sites and reducing time contact with biliopancreatic digestive secretions, micronutrient management is primordial after surgery (13, 24). Low rates of nutritional deficiencies observed in this study support the importance of a quality, long-term follow-up as offered to patients in our institution.

Before surgery, vitamin D insufficiency and iron deficiency were the most prevalent nutritional problem in all three groups. Similar or higher rates of vitamin D and iron deficiency were observed before bariatric surgery in other studies (18, 19, 33). It is not surprising that iron deficiency is prevalent in this sample since most of the participants are women, and premenopausal women are particularly affected by iron deficiency (34). For calcium, vitamin A and vitamin B12 deficiencies, rates in our groups were below 5%. While these rates are lower than those observed in other studies (19, 33), Peterson et al. observed similar rates before RYGB (18). The lower rates of deficiency present in our groups at baseline may explain why we observed less deficiencies than previous literature in the postoperative period. Indeed, nutritional deficiencies prior to surgery are an important risk factor for developing nutritional problems after surgery (12), highlighting the importance of addressing them with adequate nutritional assessment and management prior to surgery. Furthermore, rates of vitamin D and iron deficiency were lower 24 months after surgery than at baseline. Our medical and nutritional care sequence not only prevented nutritional deficiencies after bariatric surgery, but also addressed pre-existing concerns for many patients in our sample. Comparable deficiency rates at baseline were observed in a previous retrospective study by our group (30). Yet, these results are at variance with literature on SG, RYGB and BPD-DS, where micronutrient deficiency rates at baseline remained stable or increased after the procedures (25, 35, 36).

### 4.2 Surgical implications

Serum levels of vitamin D were higher after surgery compared to baseline in all surgical groups. Also, mean levels of all groups were in the normal range during the entire follow-up. However, a decrease in vitamin D levels in the BPD-DS group was observed between 12 and 24 months. Vitamin D levels increased during the follow-up of patients correctly supplemented (37, 38). Regarding calcium, levels decreased during the entire follow-up in all groups. Even though BPD-DS had lower levels compared to SG at 24 months, rates of calcium deficiency were similar among all surgical groups at that time point. Tardio et al. showed a decrease in calcium levels 6 months after BPD-DS in a large sample (n = 1,436) (37), while a systematic review and meta-analysis reported no significant change after RYGB and an increase in calcium levels after SG (38). In our study, although elevated PTH levels were almost absent in all groups during postoperative care, PTH levels increased after bariatric surgery in our three groups. Other studies suggest that PTH levels increase after BPD-DS but remain stable after SG or RYGB (37, 39). A study by Syn et al. showed, in a large sample of Japanese men and women, that SG patients had lower vitamin D but higher calcium levels than RYGB patients at 24 months. Both groups presented similar levels of PTH in that sample at 24 months (40). Calcium and vitamin D absorption is reduced after hypoabsorptive bariatric procedures and commonly cause secondary hyperparathyroidism that can contribute to postoperative bone loss (24). Based on the low deficiency rates observed, status in vitamin D, calcium and PTH appears adequate in these groups, although evolution patterns suggest that maintaining a long-term follow-up may help prevent eventual nutritional problems and bone health deterioration.

One year after surgery, the BPD-DS group showed lower levels of hemoglobin than SG and RYGB participants and lower iron levels than SG patients only. Furthermore, iron levels increased during follow-up in all groups. Mixed results were noted regarding the effect of bariatric surgery on iron status markers with some studies showing stable levels of iron and hemoglobin after surgery (39, 41) while others found increased levels of iron and reduced levels of hemoglobin during follow-up (25, 42). In our study, ferritin levels remained stable for RYGB and BPD-DS while they decreased for SG. Some studies showed decreased ferritin levels after SG or RYGB (38, 39) but others presented increased levels after SG, RYGB or BPD-DS (41, 42). Syn et al. observed higher levels of hemoglobin in SG compared to RYGB patients while iron and ferritin levels were similar among groups at 24 months (40). Measurements of serum ferritin assess the level of iron stored in the body, dosage of hemoglobin informs on the capacity to transport oxygen in red blood cells and serum iron gives information on the adequacy of global iron supply (43). Rates of iron deficiency were low and similar among groups during the entire follow-up showing no greater risk of BPD-DS on iron status in our context of adequate supplement intake and nutritional management.

Albumin levels remained generally stable during follow-up with similar serum levels in all groups at 24 months. For prealbumin, after an early drop, levels increased in later time points to reach levels comparable to baseline for RYGB and BPD-DS or greater levels for SG. Similarly, Strain et al. observed stable albumin levels after BPD-DS (25), whereas levels were shown to decrease after SG or RYGB (30, 35, 36). Literature on prealbumin levels is inconclusive for SG and RYGB and is scarce for BPD-DS (35, 36). For both markers, levels were similar among all groups at 24 months. Albumin and prealbumin have been traditionally used as markers of nutritional status. The American Society for Parenteral and Enteral Nutrition (ASPEN) recently stated that low albumin and prealbumin serum levels are not a valid measurement of nutritional status, but rather indicators of the inflammatory status, regardless of underlying nutritional status (44). The transient reduction of prealbumin levels observed in our groups could result from acute inflammation related to surgical procedures, which is resolved in later follow-ups suggesting improved medical condition. These signs of inflammation in the early postoperative stage are only observed with prealbumin, considering its shorter half-life compared to albumin (44). Together, mean albumin and prealbumin levels were similar among groups and were in the normal range at 24 months suggesting no sign of inflammation or negative surgical evolution in the long term.

### 4.3 Supplementation adherence

Supplementation recommendations vary greatly across countries and to our knowledge, this is the first study to compare adherence to micronutrient initial supplementation recommendations for SG, RYGB and BPD-DS in a prospective design. Current literature on the topic is difficult to assess and there is a need for more long-term prospective studies, especially on hypoabsorptive procedures. We showed high adherence to vitamin and mineral initial supplementation recommendations in the first 2 years following bariatric surgery. This likely explains why we observed low prevalence of micronutrient deficiencies. While a prospective study on RYGB and SG showed an adherence rate of approximately 60% for calcium-vitamin D and vitamin B12 supplementation 2 years after the procedures (45), a systematic review and meta-analysis reported an adherence rate around 20% for the same procedures (38). Although literature shows a decrease in adherence to supplements within the first year (46), it remained stable in our sample for all supplements except calcium. Most RYGB patients in our sample took less vitamin D and multivitamin supplements than recommendations at 24 months. Additional analysis on vitamin D showed that most RYGB patients in the below-target category had normal serum levels. Instead of indicating low adherence, this showed that less vitamin D supplementation was enough to prevent deficiencies for most of these patients. Also, BPD-DS patients took significantly more vitamin A and D compared to recommendations at 24 months. Nett et al. also reported that 37.2 and 11.6% of BPD-DS patients, respectively, required higher doses of vitamin D and vitamin A supplementation within the five-year follow-up (47). Comparably to that group, we concluded that supplementation recommendations for patients undergoing BPD-DS should be revised, as they could benefit from higher initial doses of vitamin A and D.

### 4.4 Strengths and limitations

Our study has several strengths. First, we assessed for the first time the micronutrient status and adherence to vitamin and mineral initial supplementation recommendations after bariatric surgery in a prospective design and we compared 3 types of bariatric procedures: SG, RYGB, BPD-DS. Second, we evaluated the nutritional status before surgery and compared it at many time points up to 24 months. Also, the number of participants included in our study was relatively elevated in the context of a prospective study. Lastly, we presented the reference values used to determine deficiency and we evaluated the actual intake of vitamin and mineral supplements consumed by patients. Still, our study presents some limitations. This comparison of nutrient status in SG, RYGB and BPD-DS was prospective, but it was not a randomized trial due to the major differences in medical and nutritional management among procedures. Indeed, the supplementation, outcomes and risks associated with each procedure prevented randomization of the patients to the different arms of the study. Also, the data analyzed in this study are from a sample in which follow-up is ongoing. As a result, we had slightly less data available at 24 months compared to earlier time points. Our groups were also not balanced in terms of surgery type, which is representative of the proportions of the types of procedures performed in our institution. Lastly, there was a higher proportion of females in our groups and all patients were living with T2D. More studies will ascertain generalizability of our findings.

This study may help clinicians improve their practice in bariatric care. As our results showed a similar risk of developing micronutrient deficiencies for all three surgeries at 24 months, the BPD-DS appears to be a safe option regarding micronutrient status compared ot other surgeries. Also, the highest rates of micronutrient deficiencies were noted before surgery, which supports the recommendation to supplement patients in the preoperative period to prevent adverse outcomes. Using clinical experience, supplementation recommendations for patients undergoing BPD-DS should be increased, as they could benefit from higher initial doses of vitamins A and D. To improve understanding of nutritional status following bariatric surgery, micronutrient status should be evaluated for more than 24 months in a prospective design, because deficiencies may occur many years after surgery. Determinants of the nutritional risk following bariatric surgery should be investigated for early detection of patients at higher risk of developing deficiencies. Further research could also evaluate food intake to complement the characterisation of nutritional status in bariatric patients.

## 5 Conclusion

Vitamin D insufficiency was the most prevalent nutritional problem among patients before bariatric surgery. With appropriate medical and nutritional management, all surgeries led to similar rates of vitamin D, calcium, iron, vitamin A and vitamin B12 deficiency at 24 months. The metabolic advantages associated with BPD-DS could be offered to more patients as it appears to be safe regarding micronutrient status. Rates of vitamin D insufficiency and iron deficiency were lower at 24 months than at baseline, showing the importance of adequate supplementation to prevent micronutrient deficiencies and correct pre-existent ones. Adherence to vitamin and mineral initial supplementation recommendations was high in all groups after surgery, but most BPD-DS patients took vitamin A and vitamin D supplement doses above initial recommendations for this surgery. Initial vitamin A and vitamin D supplementation recommendations for BPD-DS patients should be revised upwards.

## Data availability statement

The datasets presented in this article are not readily available. The dataset may be shared upon request with approval from the local ethics committee. Requests to access the datasets should be directed to AT, andre.tchernof@criucpq.ulaval.ca.

## Ethics statement

The studies involving humans were approved by The Research Ethics Committee of the Institut universitaire de cardiologie et de pneumologie de Québec – Université Laval. The studies were conducted in accordance with the local legislation and institutional requirements. The participants provided their written informed consent to participate in this study.

## Author contributions

MC: Methodology, Investigation, Data curation, Conceptualization, Writing – review & editing, Writing – original draft, Formal analysis. LP: Data curation, Writing – review & editing, Writing – original draft, Formal analysis. MN: Methodology, Writing – review & editing, Project administration, Data curation. LB-B: Project administration, Investigation, Writing – review & editing, Resources. FJ: Project administration, Investigation, Writing – review & editing, Resources. AM: Writing – review & editing, Supervision, Investigation, Conceptualization. LB: Supervision, Resources, Writing – review & editing, Project administration, Methodology, Investigation, Funding acquisition, Conceptualization. AT: Resources, Methodology, Writing – review & editing, Supervision, Project administration, Investigation, Funding acquisition, Conceptualization.

## References

[1] Wharton S Lau DCW Vallis M Sharma AM Biertho L Campbell-Scherer D. Obesity in adults: a clinical practice guideline. CMAJ. (2020) 192:E875–91. doi: 10.1503/cmaj.191707, PMID: 32753461 PMC7828878

[2] Unick JL Beavers D Bond DS Clark JM Jakicic JM Kitabchi AE. The long-term effectiveness of a lifestyle intervention in severely obese individuals. Am J Med. (2013) 126:236–42. doi: 10.1016/j.amjmed.2012.10.010, PMID: 23410564 PMC3574274

[3] Schauer PR Kashyap SR Wolski K Brethauer SA Kirwan JP Pothier CE. Bariatric surgery versus intensive medical therapy in obese patients with diabetes. N Engl J Med. (2012) 366:1567–76. doi: 10.1056/NEJMoa1200225, PMID: 22449319 PMC3372918

[4] Angrisani L Santonicola A Iovino P Palma R Kow L Prager G. IFSO worldwide survey 2020-2021: current trends for bariatric and metabolic procedures. Obes Surg. (2024) 34:1075–85. doi: 10.1007/s11695-024-07118-3, PMID: 38438667 PMC11026210

[5] Marceau P Biron S Bourque R-A Potvin M Hould FS Simard S. Biliopancreatic diversion with a New type of gastrectomy. Obes Surg. (1993) 3:29–35. doi: 10.1381/096089293765559728 10757900

[6] Biertho L Lebel S Marceau S Hould FS Lescelleur O Marceau P. Laparoscopic sleeve gastrectomy: with or without duodenal switch? A consecutive series of 800 cases. Dig Surg. (2014) 31:48–54. doi: 10.1159/000354313 24819497

[7] Mahawar KK Sharples AJ. Contribution of malabsorption to weight loss after roux-en-Y gastric bypass: a systematic review. Obes Surg. (2017) 27:2194–206. doi: 10.1007/s11695-017-2762-y, PMID: 28585108

[8] Peterli R Wolnerhanssen BK Vetter D Nett P Gass M Borbely Y. Laparoscopic sleeve gastrectomy versus roux-Y-gastric bypass for morbid Obesity-3-year outcomes of the prospective randomized Swiss multicenter bypass or sleeve study (SM-BOSS). Ann Surg. (2017) 265:466–73. doi: 10.1097/SLA.0000000000001929, PMID: 28170356 PMC5300030

[9] Marceau P Biron S Marceau S Hould FS Lebel S Lescelleur O. Long-term metabolic outcomes 5 to 20 years after biliopancreatic diversion. Obes Surg. (2015) 25:1584–93. doi: 10.1007/s11695-015-1599-5, PMID: 25676155

[10] Biertho L Hong D Gagner M. Canadian adult obesity clinical practice guidelines: bariatric surgery: surgical options and outcomes (2020). Available at: https://obesitycanada.ca/guidelines/surgeryoptions (Accessed April 16, 2024).

[11] Arterburn DE Telem DA Kushner RF Courcoulas AP. Benefits and risks of bariatric surgery in adults: a review. JAMA. (2020) 324:879–87. doi: 10.1001/jama.2020.12567 32870301

[12] O'Kane M Parretti HM Pinkney J Welbourn R Hughes CA Mok J. British obesity and metabolic surgery society guidelines on perioperative and postoperative biochemical monitoring and micronutrient replacement for patients undergoing bariatric surgery-2020 update. Obes Rev. (2020) 21:e13087. doi: 10.1111/obr.13087, PMID: 32743907 PMC7583474

[13] Gasmi A Bjørklund G Mujawdiya PK Semenova Y Peana M Dosa A. Micronutrients deficiences in patients after bariatric surgery. Eur J Nutr. (2022) 61:55–67. doi: 10.1007/s00394-021-02619-8 34302218

[14] Mechanick JI Apovian C Brethauer S Timothy Garvey W Joffe AM Kim J. Clinical practice guidelines for the perioperative nutrition, metabolic, and nonsurgical support of patients undergoing bariatric procedures −2019 update: cosponsored by American Association of Clinical Endocrinologists/American College of Endocrinology, the Obesity Society, American Society for Metabolic and Bariatric Surgery, obesity medicine association, and American Society of Anesthesiologists. Obesity (Silver Spring). (2020) 28:O1–O58. doi: 10.1002/oby.22719 32202076

[15] Shiau J Biertho L. Canadian adult obesity clinical practice guidelines: bariatric surgery: postoperative management (2020). Available at: https://obesitycanada.ca/guidelines/postop (Accessed April 16, 2024).

[16] Kobylinska M Antosik K Decyk A Kurowska K. Malnutrition in obesity: is it possible? Obes Facts. (2022) 15:19–25. doi: 10.1159/000519503, PMID: 34749356 PMC8820192

[17] Astrup A Bugel S. Overfed but undernourished: recognizing nutritional inadequacies/deficiencies in patients with overweight or obesity. Int J Obes. (2019) 43:219–32. doi: 10.1038/s41366-018-0143-9 29980762

[18] Peterson LA Cheskin LJ Furtado M Papas K Schweitzer MA Magnuson TH. Malnutrition in bariatric surgery candidates: multiple micronutrient deficiencies prior to surgery. Obes Surg. (2016) 26:833–8. doi: 10.1007/s11695-015-1844-y, PMID: 26297429

[19] Lefebvre P Letois F Sultan A Nocca D Mura T Galtier F. Nutrient deficiencies in patients with obesity considering bariatric surgery: a cross-sectional study. Surg Obes Relat Dis. (2014) 10:540–6. doi: 10.1016/j.soard.2013.10.003, PMID: 24630922

[20] Gastrointestinal surgery for severe obesity. National institutes of health consensus development conference statement. Am J Clin Nutr. (1992) 55:615S–9S. doi: 10.1093/ajcn/55.2.615s 1733140

[21] Biertho L Simon-Hould F Marceau S Lebel S Lescelleur O Biron S. Current outcomes of laparoscopic duodenal switch. Ann Surg Innov Res. (2016) 10:1. doi: 10.1186/s13022-016-0024-7, PMID: 26807142 PMC4722734

[22] Zeighami Y Iceta S Dadar M Pelletier M Nadeau M Biertho L. Spontaneous neural activity changes after bariatric surgery: a resting-state fMRI study. NeuroImage. (2021) 241:118419. doi: 10.1016/j.neuroimage.2021.118419, PMID: 34302967

[23] Biertho L Biron S Hould FS Lebel S Marceau S Marceau P. Is biliopancreatic diversion with duodenal switch indicated for patients with body mass index <50 kg/m2? Surg Obes Relat Dis. (2010) 6:508–14. doi: 10.1016/j.soard.2010.03.285, PMID: 20627706

[24] Via MA Mechanick JI. Nutritional and micronutrient Care of Bariatric Surgery Patients: current evidence update. Curr Obes Rep. (2017) 6:286–96. doi: 10.1007/s13679-017-0271-x, PMID: 28718091

[25] Strain GW Torghabeh MH Gagner M Ebel F Dakin GF Connolly D. Nutrient status 9 years after biliopancreatic diversion with duodenal switch (BPD/DS): an observational study. Obes Surg. (2017) 27:1709–18. doi: 10.1007/s11695-017-2560-6, PMID: 28155056

[26] Kwon Y Ha J Lee YH Kim D Lee CM Kim JH. Comparative risk of anemia and related micronutrient deficiencies after roux-en-Y gastric bypass and sleeve gastrectomy in patients with obesity: an updated meta-analysis of randomized controlled trials. Obes Rev. (2022) 23:e13419. doi: 10.1111/obr.13419, PMID: 35048495

[27] Ferraz AAB Carvalho MRC Siqueira LT Santa-Cruz F Campos JM. Micronutrient deficiencies following bariatric surgery: a comparative analysis between sleeve gastrectomy and roux-en-Y gastric bypass. Rev Col Bras Cir. (2018) 45:e2016. doi: 10.1590/0100-6991e-20182016, PMID: 30540099

[28] Arias PM Domeniconi EA Garcia M Esquivel CM Martinez Lascano F Foscarini JM. Micronutrient deficiencies after roux-en-Y gastric bypass: long-term results. Obes Surg. (2020) 30:169–73. doi: 10.1007/s11695-019-04167-x, PMID: 31502183

[29] Moize V Andreu A Flores L Torres F Ibarzabal A Delgado S. Long-term dietary intake and nutritional deficiencies following sleeve gastrectomy or roux-En-Y gastric bypass in a mediterranean population. J Acad Nutr Diet. (2013) 113:400–10. doi: 10.1016/j.jand.2012.11.013, PMID: 23438491

[30] Caron M Hould FS Lescelleur O Marceau S Lebel S Julien F. Long-term nutritional impact of sleeve gastrectomy. Surg Obes Relat Dis. (2017) 13:1664–73. doi: 10.1016/j.soard.2017.07.019 29054174

[31] Gesquiere I Foulon V Augustijns P Gils A Lannoo M Van der Schueren B. Micronutrient intake, from diet and supplements, and association with status markers in pre-and post-RYGB patients. Clin Nutr. (2017) 36:1175–81. doi: 10.1016/j.clnu.2016.08.009, PMID: 27591033

[32] Cloutier A Lebel S Hould F Julien F Marceau S Bouvet L. Long alimentary limb duodenal switch (LADS): a short-term prospective randomized trial. Surg Obes Relat Dis. (2018) 14:30–7. doi: 10.1016/j.soard.2017.08.028, PMID: 29217129

[33] Sanchez A Rojas P Basfi-Fer K Carrasco F Inostroza J Codoceo J. Micronutrient deficiencies in morbidly obese women prior to bariatric surgery. Obes Surg. (2016) 26:361–8. doi: 10.1007/s11695-015-1773-9, PMID: 26108638

[34] Pasricha S-R Tye-Dyn J Muckenthaler MU Swinkels DW. Iron deficiency. Lancet. (2021) 397:233–48. doi: 10.1016/S0140-6736(20)32594-0 33285139

[35] Voglino C Tirone A Ciuoli C Benenati N Bufano A Croce F. Controlling nutritional status (CONUT) score and micronutrient deficiency in bariatric patients: midterm outcomes of roux-en-Y gastric bypass versus one anastomosis gastric bypass/Mini gastric bypass. Obes Surg. (2021) 31:3715–26. doi: 10.1007/s11695-021-05486-8, PMID: 34031850

[36] Verger EO Aron-Wisnewsky J Dao MC Kayser BD Oppert JM Bouillot JL. Micronutrient and protein deficiencies after gastric bypass and sleeve gastrectomy: a 1-year follow-up. Obes Surg. (2016) 26:785–96. doi: 10.1007/s11695-015-1803-7, PMID: 26205215

[37] Tardio V Blais JP Julien AS Douville P Lebel S Biertho L. Serum parathyroid hormone and 25-Hydroxyvitamin D concentrations before and after biliopancreatic diversion. Obes Surg. (2018) 28:1886–94. doi: 10.1007/s11695-017-3101-z, PMID: 29322299

[38] Ha J Kwon Y Kwon JW Kim D Park SH Hwang J. Micronutrient status in bariatric surgery patients receiving postoperative supplementation per guidelines: insights from a systematic review and meta-analysis of longitudinal studies. Obes Rev. (2021) 22:e13249. doi: 10.1111/obr.13249, PMID: 33938111

[39] Vinolas H Barnetche T Ferrandi G Monsaingeon-Henry M Pupier E Collet D. Oral hydration, food intake, and nutritional status before and after bariatric surgery. Obes Surg. (2019) 29:2896–903. doi: 10.1007/s11695-019-03928-y, PMID: 31102207

[40] Syn NL Lee PC Kovalik JP Tham KW Ong HS Chan WH. Associations of bariatric interventions with micronutrient and endocrine disturbances. JAMA Netw Open. (2020) 3:e205123. doi: 10.1001/jamanetworkopen.2020.5123, PMID: 32515795 PMC7284307

[41] Homan J Schijns W Aarts EO Janssen IMC Berends FJ de Boer H. Treatment of vitamin and mineral deficiencies after biliopancreatic diversion with or without duodenal switch: a major challenge. Obes Surg. (2018) 28:234–41. doi: 10.1007/s11695-017-2841-0, PMID: 28861696

[42] Shipton MJ Johal NJ Dutta N Slater C Iqbal Z Ahmed B. Haemoglobin and hematinic status before and after bariatric surgery over 4 years of follow-up. Obes Surg. (2021) 31:682–93. doi: 10.1007/s11695-020-04943-0, PMID: 32875517 PMC7847875

[43] Lynch S Pfeiffer CM Georgieff MK Brittenham G Fairweather-Tait S Hurrell RF. Biomarkers of nutrition for development (BOND)-Iron review. J Nutr. (2018) 148:1001S–67S. doi: 10.1093/jn/nxx036 29878148 PMC6297556

[44] Evans DC Corkins MR Malone A Miller S Mogensen KM Guenter P. The use of visceral proteins as nutrition markers: an ASPEN position paper. Nutr Clin Pract. (2021) 36:22–8. doi: 10.1002/ncp.10588, PMID: 33125793

[45] Spetz K Svedjeholm S Roos S Grehn S Olbers T Andersson E. Adherence to vitamin and mineral supplementation after bariatric surgery - a two-year cohort study. Obes Res Clin Pract. (2022) 16:407–12. doi: 10.1016/j.orcp.2022.09.001, PMID: 36151032

[46] Aasheim ET Bjorkman S Sovik TT Engstrom M Hanvold SE Mala T. Vitamin status after bariatric surgery: a randomized study of gastric bypass and duodenal switch. Am J Clin Nutr. (2009) 90:15–22. doi: 10.3945/ajcn.2009.27583, PMID: 19439456

[47] Nett P Borbely Y Kroll D. Micronutrient supplementation after biliopancreatic diversion with duodenal switch in the long term. Obes Surg. (2016) 26:2469–74. doi: 10.1007/s11695-016-2132-1, PMID: 26983747

