# Supplementary material for: Micronutrient status 2 years after bariatric surgery: a prospective nutritional assessment
Source: Front Nutr. 2024 May 14;11:1385510. doi: 10.3389/fnut.2024.1385510 (PMC11132185; doi:10.3389/fnut.2024.1385510)
Supplement: Supplementary file 1 [file Table_1.DOCX]

| **Supplementary Table 1. Serum references values used to determine nutritional dificiency or other sub-optimal nutritional status** | | |
| --- | --- | --- |
| **Nutritional indicator** |  | **Reference value for nutritional problem** |
| Iron |  | Female : < 9 µmol/L |
|  |  | Male: < 11 µmol/L |
| Ferritin |  | Female < 45 years : < 15 µg/L |
|  |  | Female > 45 years : < 10 µg/L |
|  |  | Male : < 25 µg/L |
| Transferrin |  | < 1.8 g/L |
| Hemoglobin |  | Female: < 120 g/L |
|  |  | Male: < 140 g/L |
| Calcium |  | < 1.14 mmol/L |
| Phosphorus |  | < 0.8 mmol/L |
| Parathormone |  | > 20 ng/L |
| Vitamin D |  | Insufficiency: 30-49 nmol/L |
|  |  | Deficiency: < 30 nmol/L |
| Vitamin A |  | < 0.7 µmol/L |
| Vitamin B12 |  | < 135 pmol/L |
| Folate |  | < 8 nmol/L |
| Sodium |  | < 135 mmol/L |
| Potassium |  | < 3.4 mmol/L |
| Chlore |  | < 98 mmol/L |
| Magnesium |  | < 0.65 mmol/L |
| Albumin |  | < 35 g/L |
| Prealbumin |  | < 200 mg/L |
| Lab references values from the Quebec Heart and Lung Institute or the Québec university hospital center | | |
